# Dual-Path Large Kernel Learning and Its Applications in Single-Image Super-Resolution †

**DOI:** 10.3390/s24196174

**Published:** 2024-09-24

**Authors:** Zhen Su, Mang Sun, He Jiang, Xiang Ma, Rui Zhang, Chen Lv, Qiqi Kou, Deqiang Cheng

**Affiliations:** 1 School of Information and Control Engineering, China University of Mining and Technology, Xuzhou 221116, China; suzhen83@163.com (Z.S.); sunmang@cumt.edu.cn (M.S.); maxiang00@cumt.edu.cn (X.M.); zrcumt@cumt.edu.cn (R.Z.); chengdq@cumt.edu.cn (D.C.); 2 Key Laboratory of System Control and Information Processing, Ministry of Education, Shanghai 200240, China; 3 School of Computer Science, China University of Mining and Technology, Xuzhou 221116, China; kouqiqi@cumt.edu.cn

**Keywords:** super-resolution, large kernel learning, dual path, lightweight

## Abstract

To enhance the performance of super-resolution models, neural networks frequently employ module stacking. However, this approach inevitably results in an excessive proliferation of parameter counts and information redundancy, ultimately constraining the deployment of these models on mobile devices. To surmount this limitation, this study introduces the application of Dual-path Large Kernel Learning (DLKL) to the task of image super-resolution. Within the DLKL framework, we harness a multiscale large kernel decomposition technique to efficiently establish long-range dependencies among pixels. This network not only maintains excellent performance but also significantly mitigates the parameter burden, achieving an optimal balance between network performance and efficiency. When compared with other prevalent algorithms, DLKL exhibits remarkable proficiency in generating images with sharper textures and structures that are more akin to natural ones. It is particularly noteworthy that on the challenging texture dataset Urban100, the network proposed in this study achieved a significant improvement in Peak Signal-to-Noise Ratio (PSNR) for the ×4 upscaling task, with an increase of 0.32 dB and 0.19 dB compared with the state-of-the-art HAFRN and MICU networks, respectively. This remarkable result not only validates the effectiveness of the present model in complex image super-resolution tasks but also highlights its superior performance and unique advantages in the field.

## 1. Introduction

Single-Image Super-Resolution (SISR) technology aims to finely recover high-resolution (HR) images with rich details from low-resolution (LR) images. This technology plays an indispensable role in various fields, such as medical image retrieval [1,2], computational photography [3,4,5,6,7], and smart mines [8]. Currently, mainstream SISR algorithms can be categorized into three types: interpolation-based methods, reconstruction-based methods, and learning-based methods. Although interpolation-based methods are favored for their fast execution, the reconstructed images often suffer from artifacts such as blur artifact, leading to unsatisfactory image quality. On the other hand, reconstruction-based methods constrain the solution space by introducing prior knowledge, which can improve the reconstruction quality to a certain extent. However, they often come with complex optimization processes and high computational costs, limiting their practical application to some extent.

In recent years, convolutional neural networks have achieved remarkable breakthroughs and advancements in the field of super-resolution technology. Among these advancements, the introduction of the SRCNN [9] stands out for its remarkable reconstruction performance, despite its succinct structure comprising merely three convolutional layers. This approach has served as the foundation for the classical three-stage framework that subsequent super-resolution models have followed: shallow feature extraction, nonlinear feature mapping, and image reconstruction. To alleviate the computational complexity of SRCNN, researchers introduced the pixel shuffle strategy and ingeniously applied it to the image reconstruction stage, thereby significantly enhancing the computational efficiency of the neural network. Subsequently, to further improve the reconstruction quality, more extensive and deeper super-resolution networks such as VDSR [10], EDSR-BASE [11], and DRCN [12] have emerged. Although there is a positive correlation between network depth and performance, deep networks often come with high training costs. Therefore, attention-based SISR networks gradually emerge, which can intelligently focus on significant information while ignoring minor details, thus achieving more accurate reconstruction. Models like RCAN [13], PAN [14], and IPT [15] are typical representatives of this approach, integrating channel attention, pixel attention, and self-attention mechanisms, respectively. However, existing attention mechanisms still face challenges in integrating local and global dependencies, and a single attention mechanism often struggles to capture diverse information. To address this issue, researchers propose methods that combine multiple attention mechanisms, such as MFRN [16], combining channel attention and pixel attention, while CBAM [17] integrates channel attention and spatial attention. Although these methods improve performance to some extent, they also significantly increase computational demands, making practical applications on resource-constrained mobile devices difficult. Therefore, achieving excellent performance while maintaining lightweight characteristics becomes a hot research topic. In recent years, researchers actively explore lightweight architectures such as LMAN [18], MSFIN [19], and MADNet [20]. Although these architectures reduce model parameters to some extent, achieving further reductions in computational complexity while maintaining performance, especially in preserving complex details, remains a challenging task.

To tackle this challenge, VAN [21] creatively proposes a novel approach called large kernel attention (LKA). This method forms a unique and efficient structural system by ingeniously combining deep convolution, deep dilated convolution, and pointwise convolution. Although LKA performs well in terms of computational efficiency, it has limitations in capturing and utilizing multivariate information due to its reliance on fixed kernel sizes. To address these limitations, this study further combines the multiscale mechanism with LKA, innovatively proposing the Dual-path Large Kernel Learning (DLKL) method. DLKL not only captures long-range dependencies in images but also effectively preserves local detailed information, enabling fine adjustments of image textures. Additionally, this method significantly enhances the acquisition of spatial diversity information while effectively suppressing redundant data, thereby improving processing efficiency. Experimental results demonstrate that DLKL outperforms mainstream algorithms in both objective metrics and visual perception. Importantly, compared with other lightweight algorithms, DLKL achieves a significant reduction in parameter count while maintaining excellent performance, thus achieving a perfect balance between model performance and efficiency. In summary, the contributions of this study mainly lie in the following three aspects:

- An architecture called DLKL has been proposed.
- A dual-path large kernel attention mechanism is designed based on DLKL.
- A lightweight SISR model employing the DLKL method is introduced.

## 2. Proposed Method

**Figure 1:**
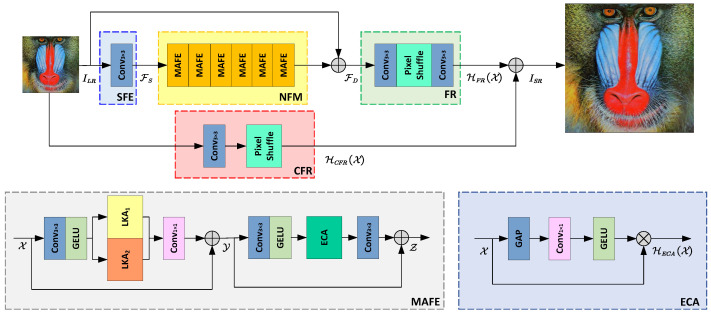
Flowchart of the DLKL system, where the symbol ⊕ means element-wise sum.

Figure 1 provides a detailed illustration of a SISR system based on Dual-path Large Kernel Learning (DLKL). The system consists of four components: shallow feature extraction (SFE), nonlinear feature mapping (NFM), feature reconstruction (FR), and coarse feature reconstruction (CFR). It should be clear that each module in the system plays an indispensable role. Specifically, the SFE module, as the feature coarse extraction module, provides the necessary foundation for the subsequent fine texture extraction. The NFM module, i.e., the nonlinear mapping module, is responsible for utilizing the linear and nonlinear features to achieve the accurate fitting of the fine texture. And the FR and CFR modules, as pixel shuffling modules, not only achieve image resolution enhancement but also undertake the recovery of fine-grained and coarse-grained image details.

### 2.1. Shallow Feature Extraction (SFE)

Assuming the input low-resolution image is $I_{LR}$, this image will be processed through the shallow feature extraction function $H_{SFE}$ to obtain the shallow feature $F_{S}$. This process can be described by Equation (1).

(1) $$F_{S}=H_{SFE}\left(I_{LR}\right)$$

where $I^{LR}$ represents the low-resolution image, $H_{conv3}\left(\right)$ denotes the convolution operation with a 3×3 kernel, and $F_{S}$ represents the extracted shallow features.

Due to the limitations of 1×1 convolutions in capturing spatial dependencies, they often result in significant loss of low-frequency information in applications. On the other hand, while 5×5 convolutions have a larger receptive field, they may also introduce a significant amount of task-irrelevant information, thereby increasing the complexity of the network. In contrast, 3×3 convolutions maintain spatial correlations while reducing the number of parameters, thus achieving network lightweighting. Therefore, in the shallow feature extraction function $H_{SFE}$, a solitary 3×3 convolutional layer is exclusively employed. For a given tensor X, the process can be described using Equation (2), and $C_{3\times 3}$ denotes a 3×3 convolutional kernel.

(2) $$H_{SFE}\left(X\right)=C_{3\times 3}\left(X\right)$$

### 2.2. Nonlinear Feature Mapping (NFM)

Subsequently, the extracted shallow features are passed to a deep feature extraction network formed by cascading multiple MAFE modules. This processing is described by Equation (3), where $H_{MAFE}^{6}\left(\cdot \right)$ represents the continuous propagation function of six MAFE modules, and $F_{D}$ refers to the deep features obtained from the deep feature extraction network.

(3) $$F_{D}=H_{MAFE}^{6}\left(I_{LR}\right)⊕I_{LR}$$

#### 2.2.1. MAFE

MAFE stands for Multiscale Attention Feature Extraction. In order to achieve good super-resolution performance with a low number of parameters, this paper designs a lightweight SISR network. In this network, the article innovatively combines large kernel decomposition, multiscale learning, and channel attention enhancement to address the challenges in super-resolution tasks. Specifically, MAFE includes the following three core aspects: firstly, it can establish interdependence between features in the spatial dimension, thereby enhancing the correlation between features; secondly, MAFE can capture feature information at different scales, improving the network’s ability to process the complex image content while maintaining efficiency; finally, by introducing channel attention mechanism, MAFE significantly elevates the expression capability of features in each channel, thereby allowing the network to concentrate more intently on the pivotal features that are indispensable for super-resolution reconstruction.

MAFE consists of multiple convolutional layers, two Gaussian Error Linear Unit (GELU) activation functions, the LKA mechanism, and the Enhanced Channel Attention (ECA) module [22]. Given the input tensor X, the processing flow of MAFE can be described by Equations (4) and (5). In these equations, Y and Z represent the generated features, δ(·) denotes the GELU activation function used to introduce nonlinearity to the network, cat(·) is a versatile feature concatenation function that effectively integrates features from various sources to enhance model performance, and $H_{LKA}$ and $H_{ECA}$ are the propagation functions of the LKA and ECA mechanisms, respectively, used to capture long-range dependencies and enhance the discriminative power of channel features, while $C_{1\times 1}$ is a 1×1 convolutional kernel used to adjust the dimensionality of features.

(4) $$\begin{array}{c} Y=C_{1\times 1}\left(cat\left[H_{LKA1}\left(\delta \left(C_{3\times 3}\left(X\right)\right)\right),H_{LKA2}\left(\delta \left(C_{3\times 3}\left(X\right)\right)\right)\right]\right))⊕X \end{array}$$

(5) $$Z=C_{3\times 3}\left(H_{ECA}\left(\delta \left(C_{3\times 3}\left(Y\right)\right)\right)\right)⊕Y$$

#### 2.2.2. LKA

**Figure 2:**
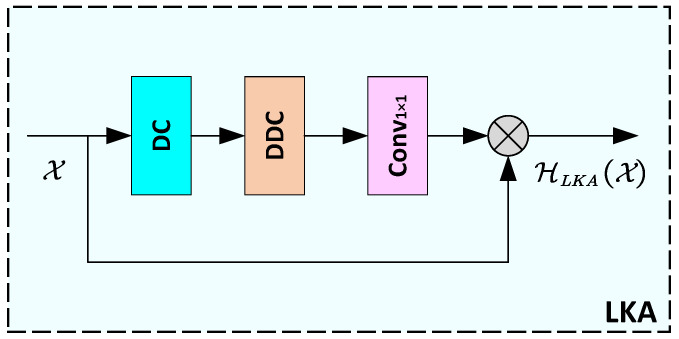
Structure of LKA, where DC means depth convolution and DDC means depth-dilated convolution.

Given the input feature X, the LKA mechanism ingeniously decomposes the convolution into three key components, thus establishing long-range dependencies in the image. As shown in Figure 2, these components include a (2d−1)×(2d−1) depth convolution $H_{DC}\left(\cdot \right)$, a K/d×K/d depth-dilated convolution $H_{DDC}\left(\cdot \right)$, and a point-wise convolution $C_{1\times 1}\left(\cdot \right)$. The input feature first passes through the depth convolution layer, then through the depth dilated convolution layer, and finally through the point-wise convolution layer, resulting in the generation of a precise attention map. This attention map is subsequently element-wise multiplied with the original feature map, highlighting the crucial feature regions for super-resolution reconstruction, and generating a new enhanced feature map. The entire process is depicted by Equation (6), where ⊗ denotes the element-wise multiplication operation.

(6) $$H_{LKA}\left(X\right)=C_{1\times 1}\left(H_{DDC}\left(H_{DC}\left(X\right)\right)\right)⊗X$$

In this paper, two sets of LKA mechanisms are designed. Among them, the LKA with a larger receptive field focuses on capturing long-range information dependencies in the image, while the LKA with a smaller receptive field emphasizes preserving local texture details. Compared with common attention methods, the proposed LKA mechanism integrates the advantages of multiscale mechanisms, considering not only the dynamic fusion process of local semantic information but also reducing the channel dimensionality through pointwise convolution, thereby achieving adaptive adjustment of the spatial dimension.

### 2.3. Feature Reconstruction (FR)

**Figure 3:**
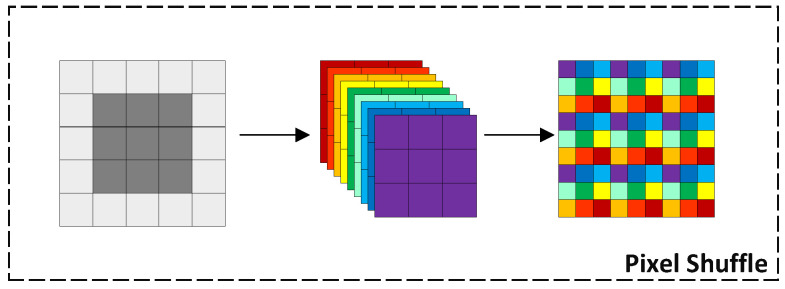
Schematic diagram of pixel shuffle.

The feature reconstruction stage follows a classical structural design, consisting of a series of cascaded 3×3 convolutional layers and pixel shuffle layers. This process can be represented by Equation (7), where $H_{FR}\left(\cdot \right)$ and $H_{PS}\left(\cdot \right)$ denote the propagation functions of feature reconstruction and pixel shuffle, respectively. They work together to achieve precise reconstruction of the input feature tensor X. The principle of subpixel convolution is shown in Figure 3.

(7) $$H_{FR}\left(X\right)=C_{3\times 3}\left(H_{PS}\left(C_{3\times 3}\left(X\right)\right)\right)$$

### 2.4. Coarse Feature Reconstruction (CFR)

The shallow features carry rich detail information, which is crucial for SISR tasks. In order to fully utilize these details, this paper carefully designs a dual-path reconstruction architecture aimed at integrating features from different paths to generate high-quality reconstructed images. In the coarse feature reconstruction path of the dual-path, we only employ a 3×3 convolutional layer and a pixel shuffle layer to ensure efficient and accurate extraction and utilization of shallow features. This process is precisely expressed by Equation (8), where $H_{CFR}\left(\cdot \right)$ and $H_{PS}\left(\cdot \right)$ represent the propagation functions of coarse feature reconstruction and pixel shuffle, respectively, and X is the input feature tensor. Finally, the $H_{FR}\left(X\right)$ obtained from feature reconstruction is added to the $H_{CFR}\left(X\right)$ obtained from coarse feature reconstruction to obtain the final reconstructed high-definition image $I_{SR}$, which can be represented by Equation (9).

(8) $$H_{CFR}\left(X\right)=H_{PS}\left(C_{3\times 3}\left(X\right)\right)$$

(9) $$I_{SR}=H_{FR}\left(X\right)⊕H_{CFR}\left(X\right)$$

### 2.5. Loss Function

In terms of optimization strategy, this paper adopts the $L_{1}$ loss function, which exhibits high sensitivity to small variations in data and helps the model converge faster. Specifically, this optimization process is explicitly demonstrated by Equation (10). In this equation, θ represents the set of parameters that need to be learned in the entire model, *N* denotes the total number of training samples, and $I_{SR}$ and $I_{HR}$, respectively, represent the images reconstructed by the model and the corresponding high-resolution ground truth images.

(10) $$L_{1}\left(\theta \right)=\frac{1}{N}\sum_{i=1}^{N}\left|\right|I_{SR}^{i}-I_{HR}^{i}{\left|\right|}_{1}$$

## 3. Experimental Results and Analysis

### 3.1. Datasets

In this paper, the DIV2K dataset is selected as the training set to ensure that the model receives sufficient training and optimization. During the testing phase, standardized benchmark datasets are employed to comprehensively evaluate the performance of different models, including Set5 [23], Set14 [24], BSD100 [25], and Urban100 [26].

### 3.2. Experimental Details

This paper adopts PyTorch 1.6 as the deep learning framework and leverages the powerful computational capabilities of the GeForce RTX 3090 GPU (NVIDIA, Santa Clara, CA, USA) for model training and testing. To enhance the model’s generalization ability, rotation and mirroring operations are used for data augmentation, enriching the diversity of the dataset. During the training process, the specific parameter settings are as follows: the batch size is set to 16, and the size of input image patches is 96×96 to ensure that the model can fully learn image features. Adam optimizer is chosen as the optimizer, with the key parameters $\beta_{1}=0.9$ and $\beta_{2}=0.999$. The initial learning rate is set to $10^{-4}$ to accelerate the convergence speed of the model in the early stage of training. When the number of model iterations reaches $2\times 10^{5}$, the learning rate is halved in a timely manner to ensure that the model can perform finer adjustments in the later stage. In order to comprehensively and objectively evaluate the performance of the model, peak signal-to-noise ratio (PSNR) and structural similarity (SSIM) are selected as the two quantitative evaluation metrics.

### 3.3. Quantitative Analytics

This study primarily employs two metrics, PSNR and SSIM, to evaluate the quality of image reconstruction. As a widely recognized image quality assessment method, PSNR accurately measures the pixel differences between the reconstructed image and the original image. Meanwhile, SSIM focuses on assessing the structural similarity of the image. In our objective quality evaluation experiment, only the y-channel component of the image was tested. The specific calculation methods for PSNR and SSIM can be found in Equations (11) and (12). In these two equations, m×n represents the size of the image, where *m* and *n* represent the number of rows and columns of the image, respectively. $I_{HR}$ stands for the ground truth image, and $I_{SR}$ represents the reconstructed image. $\mu_{x}$ and $\mu_{y}$ represent the pixel means of $I_{HR}$ and $I_{SR}$, respectively, while $\delta_{x}^{2}$ and $\delta_{y}^{2}$ represent their pixel variances, and $\delta_{xy}$ represents the pixel covariance between $I_{HR}$ and $I_{SR}$. Additionally, to avoid zero denominators, extremely small constants $c_{1}$ and $c_{2}$ are introduced, and their values are set to 0.001 in this study.

(11) $$\mathrm{PSNR}=20\log_{10}\frac{255\times mn}{\sum_{i=1}^{m}\sum_{j=1}^{n}{\left(I_{HR}\left(i,j\right)-I_{SR}\left(i,j\right)\right)}^{2}}$$

(12) $$\;\mathrm{SSIM}\;=\frac{\left(2\mu_{x}\mu_{y}+c_{1}\right)\left(2\delta_{xy}+c_{2}\right)}{\left(\mu_{x}^{2}+\mu_{y}^{2}+c_{1}\right)\left(\delta_{x}^{2}+\delta_{y}^{2}+c_{2}\right)}$$

Table 1 compares quantitative metrics for lightweight SISR models across multiple datasets, with the best results in bold and the second-best underlined. The DLKL model consistently outperforms others across all four benchmark datasets, especially at a ×4 scale. This highlights the DLKL model’s superior performance, stability, and ability to handle complex textures and detailed recovery.

### 3.4. Visual Perception

**Figure 4:**
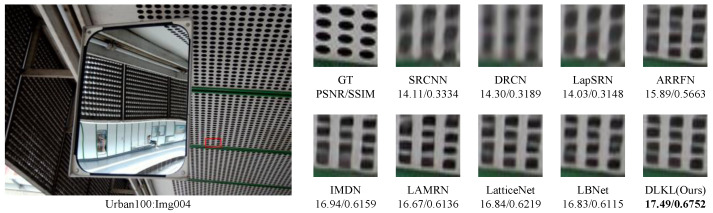
Comparison of ×4 scale reconstruction results for different algorithms on Img004 in Urban100.

**Figure 5:**
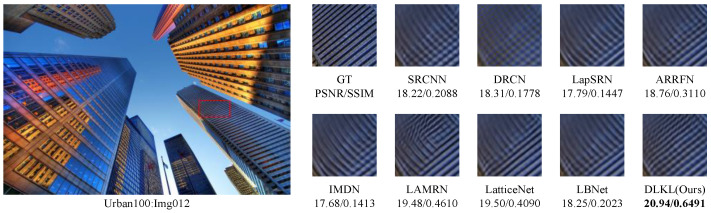
Comparison of ×4 scale reconstruction results for different algorithms on Img012 in Urban100.

**Figure 6:**
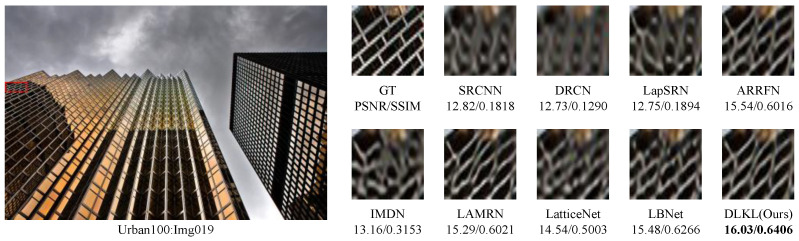
Comparison of ×4 scale reconstruction results for different algorithms on Img019 in Urban100.

**Figure 7:**
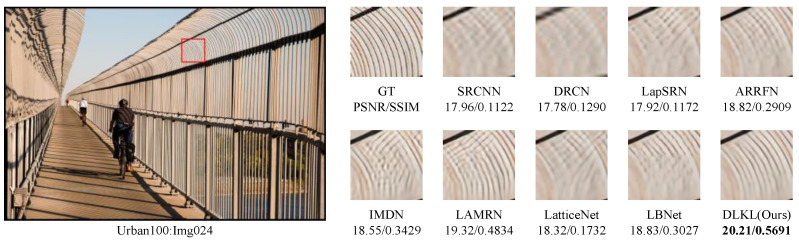
Comparison of ×4 scale reconstruction results for different algorithms on Img024 in Urban100.

**Figure 8:**
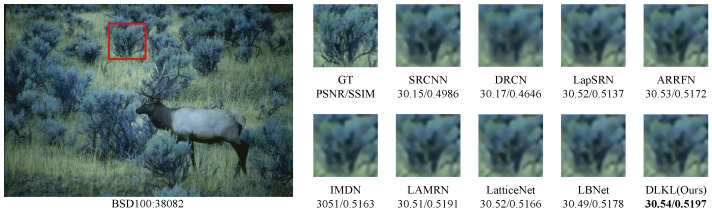
Comparison of ×4 scale reconstruction results for different algorithms on 38082 in BSD100.

**Figure 9:**
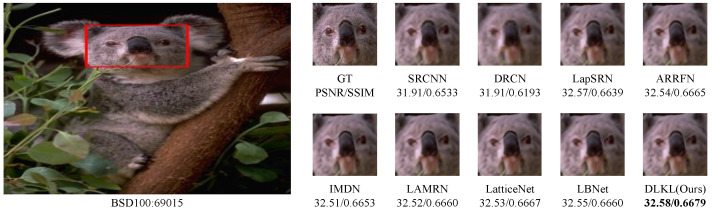
Comparison of ×4 scale reconstruction results for different algorithms on 69015 in BSD100.

To intuitively compare the reconstruction performance of various models and highlight their differences in different reconstruction effects, this study deliberately selected the Urban100 dataset with extremely high texture complexity, reconstructed the images at a ×4 magnification factor, and conducted in-depth analysis through visual effect comparisons, as shown in Figure 4, Figure 5, Figure 6, Figure 7, Figure 8 and Figure 9.

Figure 4 presents the results of reconstructing the Img004 image from the Urban100 dataset using different methods. From the figures, it can be seen that the reconstructed images of the SRCNN [9], DRCN [12], and LapSRN [27] models all exhibit significant blurring effects, showing considerable differences from the original image. The images reconstructed by the ARRFN [32] and IMDN [28] models suffer from unclear textures due to pixel loss, with adjacent black dots connected due to blurring. The LBNet [31] and LatticeNet [33] models exhibit excessive distortion in lines. In contrast, our proposed DLKL model performs better in retaining the overall shape and clarity of the black dots in the image. Figure 5 shows the reconstruction comparison of the Img012 image from the Urban100 dataset. Observing the reconstructed images produced by various algorithms, it is evident that the SRCNN [9], DRCN [12], LapSRN [27], and ARRFN [32] models lack detail processing, resulting in blurry textures. The IMDN [28], LAMRN [39], and LBNet [31] models exhibit a large number of water ripples, causing the reconstructed architectural lines to distort and be inconsistent in direction. In contrast, our DLKL model demonstrates superior subjective visual performance, with clear architectural details in the reconstruction and natural lines without distortion. Figure 6 compares the reconstruction results of the building Img019 in the Urban100 dataset. The reconstructed images of SRCNN [9], DRCN [12], IMDN [28], and LatticeNet [33] all show deficiencies in the clarity of window frame contours, background blurriness, and texture details. The ARRFN [32] and LBNet [31] models have improved in window frame details and line distortion, but the overall image is slightly dim and accompanied by slight distortion. In contrast, in our reconstruction, the DLKL model exhibits clear window frame contours, significantly improved overall clarity, and closer resemblance to the original image. Figure 7 further demonstrates the reconstruction effects of other images in the Urban100 dataset. Through comparison, we can see that our proposed DLKL algorithm significantly alleviates the overall blurring issue of the reconstruction images produced by SRCNN [9], DRCN [12], and LapSRN [27]. The images reconstructed by LatticeNet [33] and LBNet [31] exhibit line distortion, while those reconstructed by IMDN [28] and LAMRN [36], although relatively clear in railing lines, have obvious horizontal ripples in the upper part of the image. In contrast, the images reconstructed by the DLKL model not only retain the structural texture of the railing but also have clearer and more prominent lines. In summary, through subjective visual evaluation, our DLKL model demonstrates excellent performance in texture details, line authenticity, and structural clarity of reconstructed images, producing reconstructions with richer details, more natural lines, and clearer structures.

To verify the robustness of the model, we simulate not only regular texture blocks but also irregular ones, such as animal fur and grasses. Figure 8 and Figure 9 display the results for two images from the BSD100 dataset. Despite the geometric irregularity of these image blocks, our algorithm consistently achieves excellent results in quantitative metrics, namely PSNR/SSIM, demonstrating its strong performance and generalization ability.

**Figure 10:**
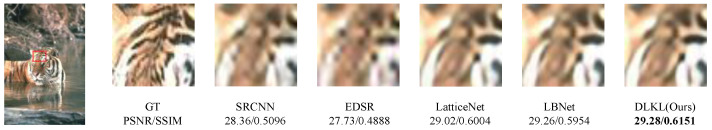
Comparison of ×4-scale reconstruction results for different algorithms on tiger.

**Figure 11:**
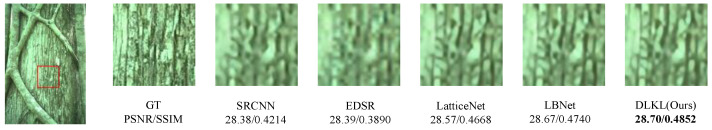
Comparison of ×4-scale reconstruction results for different algorithms on tree.

**Figure 12:**
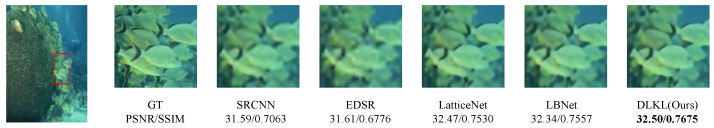
Comparison of ×4 -cale reconstruction results for different algorithms on fish.

Given that it is difficult for the benchmark dataset to fully reflect the diversity and complexity of real-world images, we further apply the DLKL algorithm to image testing in real-world scenarios, which are demonstrated in Figure 10, Figure 11 and Figure 12. Through careful observation, especially the hairs of the tiger in Figure 10, the epidermis of the tree in Figure 11, and the schools of fishes in Figure 12, we validate the applicability of the DLKL algorithm on real images. The algorithm not only effectively captures and recovers the complex and fine elements in the image, but also maintains a high degree of visual fidelity, which is a strong proof of the utility and effectiveness of the DLKL algorithm in the field of super-resolution technology.

### 3.5. Reconstruction Error

**Figure 13:**
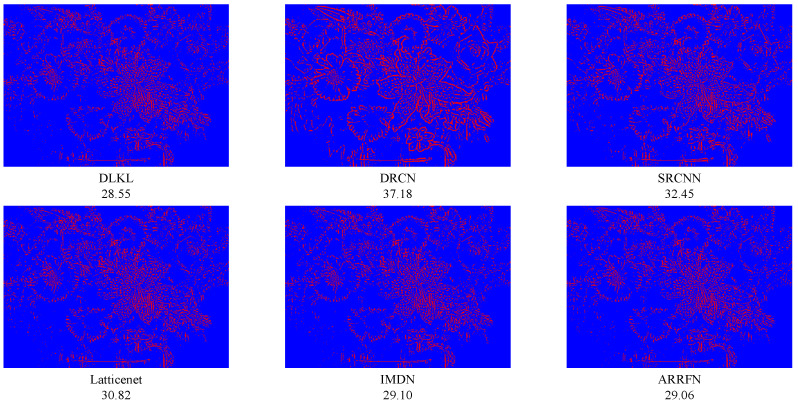
Comparison of ×4-scale reconstruction errors for different algorithms on flowers in Set14, with the name of the algorithm and its mean square error value below the image.

**Figure 14:**
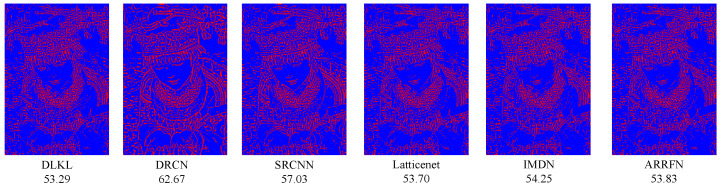
Comparison of ×4-scale reconstruction errors for different algorithms on comic in Set14, with the name of the algorithm and its mean square error value below the image.

**Figure 15:**
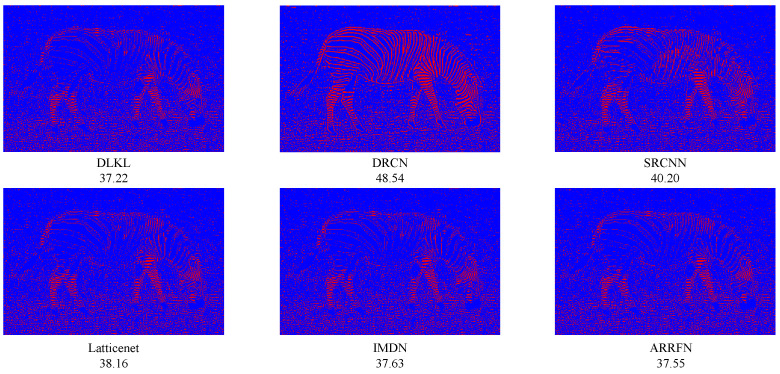
Comparison of ×4-scale reconstruction errors for different algorithms on zebra in Set14, with the name of the algorithm and its mean square error value below the image.

In order to clearly demonstrate the superiority of our method (DLKL) in terms of reconstruction accuracy, we systematically compare it with a series of frontier models (SRCNN [9], DRCN [12], IMDN [28], ARRFN [32], LatticeNet [33]). The evaluation is based on the key metric of reconstruction error, specifically using the mean square error to measure the closeness between the super-resolution images generated by each model and the real image. The results show that the reconstruction error of DLKL is significantly lower than that of all compared models, reaching the lowest level. In order to visualize this advantage, we innovatively apply a pseudocoloring technique to process the reconstruction error map, where the pixel regions with error values lower than 15 are marked in blue to indicate the low error part. As shown in Figure 13, Figure 14 and Figure 15, the red pixels (indicating high error regions) in the error maps of SRCNN [9] and DRCN [12] are more significant compared with DLKL, while the mean square errors of the other models are also higher than those of DLKL. This finding, both through visual intuitive comparison and quantitative data analysis, solidly verifies the superior performance of DLKL in reducing reconstruction errors.

**Figure 16:**
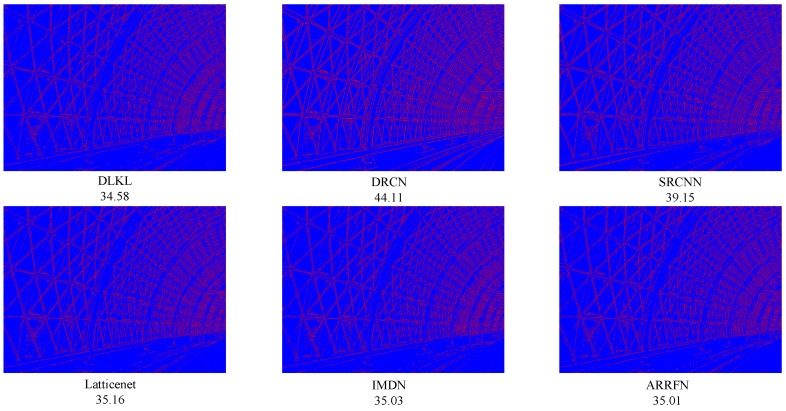
Comparison of ×4-scale reconstruction errors for different algorithms on img008 in Urban100, with the name of the algorithm and its mean square error value below the image.

**Figure 17:**
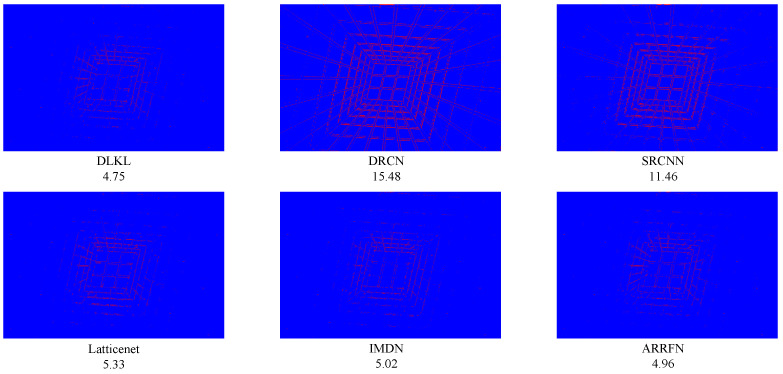
Comparison of ×4-scale reconstruction errors for different algorithms on img090 in Urban100, with the name of the algorithm and its mean square error value below the image.

**Figure 18:**
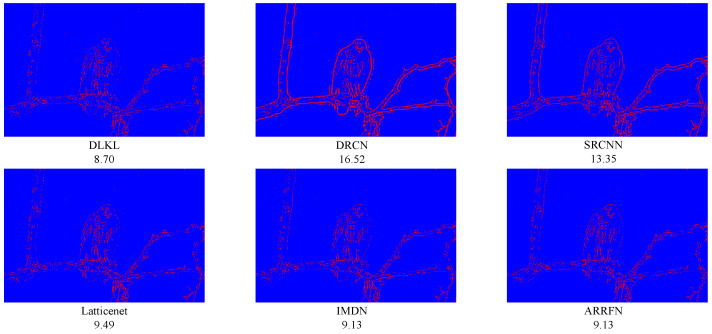
Comparison of ×4-scale reconstruction errors for different algorithms on 42049 in BSD100, with the name of the algorithm and its mean square error value below the image.

**Figure 19:**
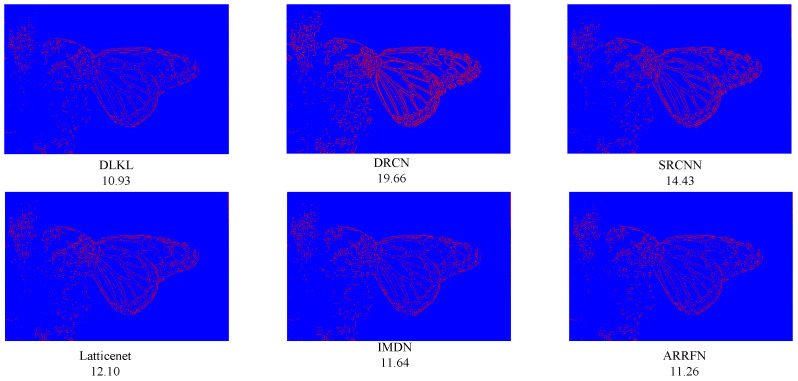
Comparison of ×4-scale reconstruction errors for different algorithms on monarch in Set14, with the name of the algorithm and its mean square error value below the image.

To verify the advantages of the DLKL algorithm in accurately processing complex texture details, we planned and implemented four special tests, each targeting a different type of complex texture image: two groups of tests focused on complex texture features in the field of modern architecture, and the other two focused on the rendering of complex textures in natural environments. The results of the experiments are presented in Figure 16, Figure 17, Figure 18 and Figure 19. Quantitative analysis shows that the DLKL algorithm achieves the lowest level of mean square error (MSE) on all texture images involved in the tests, a key metric that provides strong evidence of the algorithm’s superior performance in maintaining image quality. At the visual evaluation level, compared with algorithms such as SRCNN [9] and DRCN [12], the DLKL algorithm achieves a significant reduction in error during the image reconstruction process, as evidenced by the drastic reduction in the originally significant red error highlighted areas in the image. This significant visual improvement again reinforces the extraordinary capability and efficiency of the DLKL algorithm in the field of complex natural texture and fine architectural texture restoration.

### 3.6. MOS Comparisons

The Mean Opinion Score (MOS) stands as a widely adopted subjective evaluation metric within the domain of visual tasks, enjoying broad international usage. This metric necessitates the selection of a representative cohort of individuals, encompassing both experts and nonexperts in suitable proportions, to solicit their assessments of the provided images.

Evaluation criteria predominantly hinge on the visual comfort experienced by human observers during the image observation process. To ensure the accuracy and fairness of the evaluation, extreme scores are first excluded, and then the remaining scores are averaged in descending order to derive the final MOS result. As depicted in Table 2, our algorithm consistently demonstrates outstanding performance across most scenarios, securing the top rank in MOS assessments. Furthermore, it attains second-place performance in select contexts. This outcome not only furnishes substantial evidence of our DLKL’s capability to generate visually pleasing and natural images but also underscores its robustness and proficiency in effectively restoring a diverse array of textures present in the dataset.

### 3.7. Comparisons with the Benchmark Model IMDN

**Figure 20:**
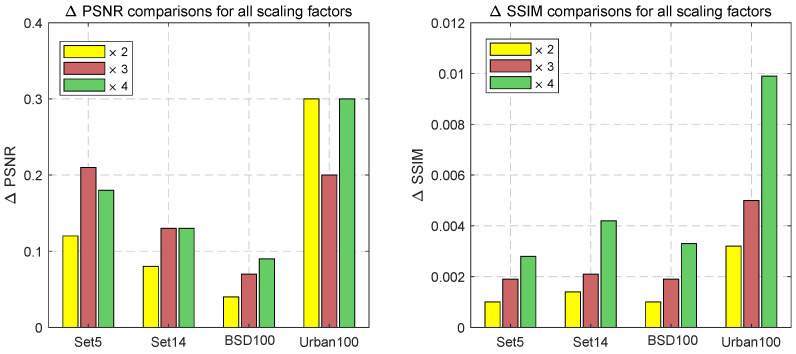
Comparison with the IMDN model. ×2, ×3 and ×4 tests in Set5, Set14, BSD100, and Urban100. Objective evaluation metrics are ∆PSNR or ∆SSIM. The test results are displayed as histograms, with the dataset names directly below.

IMDN [28] is widely regarded as the benchmark algorithm for SISR, with its effectiveness assessed using quantitative metrics such as PSNR and SSIM. The histogram in Figure 20 illustrates the ∆PSNR or ∆SSIM values between our DLKL and the IMDN benchmarks. The results demonstrate that DLKL consistently surpasses IMDN across all four internationally recognized texture datasets, particularly in more challenging ones such as BSD100 and Urban100. These findings firmly establish DLKL’s superior performance over IMDN in texture restoration.

### 3.8. Comparisons with the Recurrent-Based Methods

**Figure 21:**
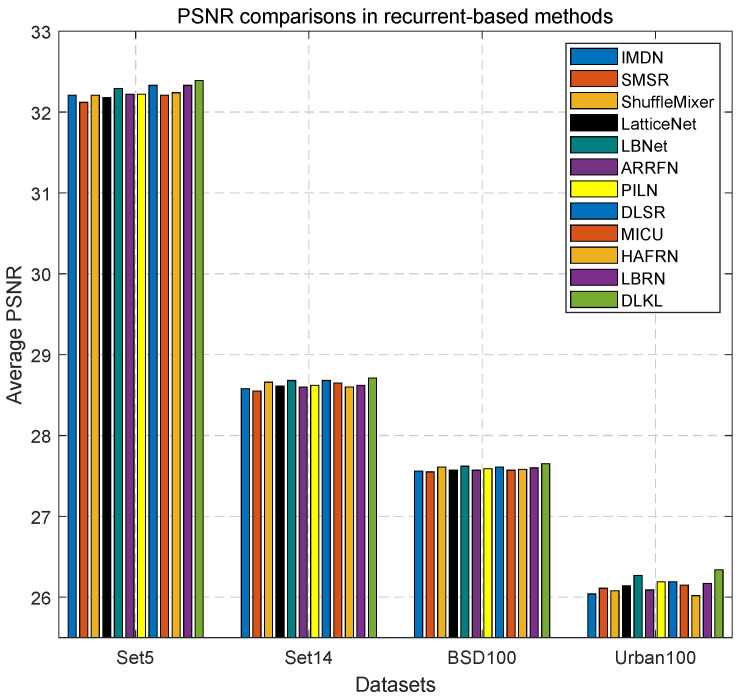
×4 SISR performance comparisons of recurrent-based algorithms. The objective evaluation metric is PSNR, and Set5, Set14, BSD100, and Urban100 are test datasets.

Since our DLKL is a recurrent-based method, presenting a concise overview of the recursive algorithm is essential. Recurrent algorithms are specifically designed to minimize training time by facilitating module reuse and effectively reducing feature loss during information transmission, thereby ensuring optimal information utilization across the entire network. To ensure a fair comparison, we benchmark our DLKL against eleven prominent SISR networks: IMDN [28], SMSR [29], ShuffleMixer [30], LatticeNet [33], LBNet [31], ARRFN [32], PILN [16], DLSR [35], MICU [36], HAFRN [37], and LBRN [38]. The evaluation results, depicted in Figure 21, clearly indicate that our DLKL achieves the highest reconstruction performance among all considered networks.

### 3.9. Comparison with Transformer-Based Methods

In recent years, researchers have started applying the transformer model to the field of image super-resolution reconstruction, achieving remarkable results. To comprehensively evaluate the performance of the DLKL model in this domain, this study specifically selects four transformer-based lightweight networks for comparison: LBNet [31], ESRT [40], NGSwin [40], and our self-developed DLKL. Detailed comparison results are presented in Table 3. To ensure fairness and accuracy, this study uses Set5, an internationally recognized and widely representative dataset, for rigorous testing. The test results indicate that the DLKL model successfully achieves optimal reconstruction results while maintaining a lightweight framework. This outcome demonstrates DLKL’s ability to efficiently recover image details during reconstruction and its stronger capacity to handle complex textures.

### 3.10. Comparisons of Topologies of Information Flow

In the design of network architectures, the connection patterns between network modules exhibit diverse characteristics. We can view these modules as hubs of information flow, whose connection layouts spatially form distinct topological structures that determine unique ways of information computation and propagation, further resulting in diverse feature expressions.

**Figure 22:**
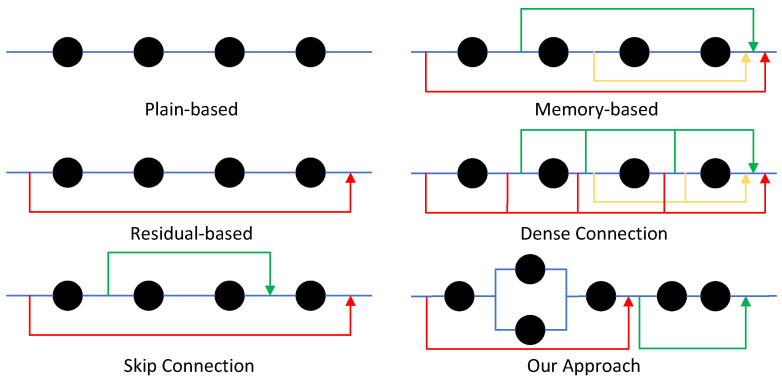
Topologies of information flow, and they are plain-based, residual-based [42], skip connection [43], memory-based [44], dense connection [45], and our approach.

In Figure 22, five typical information flow topologies are clearly presented. Among them, the black circular nodes symbolize the key nodes of information flow, while the blue lines depict the paths of information flow. Each topology has its unique information reuse mechanism, thus giving them distinct characteristics. Although skip connection networks significantly enhance algorithm efficiency by reducing computational complexity and the number of parameters, they may also lead to issues such as information loss and insufficient feature fusion. Dense connection networks greatly facilitate gradient propagation and feature reuse by directly connecting each layer to all previous layers, which is highly beneficial for training deep networks. However, this connection pattern also poses challenges such as a large number of parameters, high memory consumption, and increased computational complexity. In contrast, our method, through the ingenious introduction of dual-path reconstruction strategies, can more effectively integrate feature information, reduce the number of parameters, lower information loss, and better fuse shallow features, thus improving the quality of image reconstruction.

### 3.11. Comparisons of the Inference Speed

The inference speed of neural network models plays a crucial role in enhancing user experience, cutting costs, enabling real-time decision making, boosting deployment flexibility, and sharpening business competitiveness. Hence, during the development and optimization of models, inference speed is a vital metric that cannot be overlooked. This paper compares the reasoning speed of various common algorithms, and as evident from the Table 4, the reasoning speed presented in this paper is notably fast, further underscoring the significance of the large kernel technology employed. In this paper, we choose Urban100 as the test dataset, which contains multiple images with high-definition resolution. To ensure fairness, we adopt the average speed of processing these 100 images as the final inference speed evaluation index of the model.

### 3.12. Ablation Study

To validate the rationality of the LKA attention module proposed in this paper, we design seven sets of ablation experiments for verification. These experiments focus on different scales of LKA and multipath strategies and are evaluated using PSNR and SSIM metrics under various scale factors. Each experiment consists of four feature extraction basic blocks proposed in this paper, which are trained for a total of 400 epochs, as shown in Table 5. Initially, we investigate the influence of different sizes of LKA on the quality of reconstructed images. As the size of LKA increases, PSNR initially increases and then decreases on datasets Set5, Set14, BSD100, and Urban100. This trend occurs because both long-distance information correlation and local texture information are equally important and indispensable in image super-resolution reconstruction tasks. Subsequently, we explore the impact of multipath mechanisms on its performance. As the number of paths increases, the performance of the module improves. By considering the computational complexity, operation volume, and performance comprehensively, we ultimately select the dual-path LKA with sizes 3-5-1 and 5-7-1 as our multipath large kernel attention model.

The data presented in Table 6 provide evidence that augmenting the number of MAFEs or channels can effectively enhance the performance of the network model, albeit at the cost of increased parameter count. When holding the number of MAFEs constant, a substantial improvement in model reconstruction performance can be achieved by expanding the number of channels. However, this improvement comes with a significant surge in parameter count and computational requirements. Conversely, solely increasing the number of MAFEs results in a relatively modest rise in parameter count and computational demands. Taking into account the requirements of a lightweight super-resolution reconstruction network model, the proposed DLKL model strikes a fine balance by employing six MAFEs and setting the channel count to 64, effectively optimizing both model reconstruction performance and computational overhead.

To assess the influence of each component of MAFE on model performance, this paper conducts four sets of ablation experiments, with the results elaborated in Table 7. Initially, MAFE is solely composed of convolutional layers and GELU demonstrates subpar performance. However, upon incorporating LKA and ECA [22], notable improvements are observed in PSNR and SSIM across all four datasets. This indicates the effective capture of long-range information and intricate textures during image reconstruction, ultimately leading to the retention of both attention mechanisms in the final DLKL model.

To verify the effectiveness of the feature extraction module MAFE proposed in this paper, five sets of ablation experiments were designed. These experiments were conducted under the same experimental conditions and compared with the standard residual block (Resblock) [42], residual-in-residual dense block (RRDB) [46], Residual Channel Attention Block (RCAB) [13], and multiattention block (MAB) [47] implemented using the same LKA. The experimental results are shown in Table 8. The results indicate that the reconstruction performance of the MAFE proposed in this paper is significantly better than the other four modules while using fewer parameters and computational resources. Compared with the commonly used RRDB module in the super-resolution field, the MAFE module proposed in this paper achieved a PSNR improvement of 0.12 dB on the Set5 dataset and 0.43 dB on the challenging Urban100 dataset, which is rich in texture information. These experimental results demonstrate that the MAFE module proposed in this paper achieves a good balance between parameters, computation, and performance.

Finally, to assess the effectiveness of the dual-path reconstruction strategy, ablation experiments are conducted, and the results are presented in Table 9. The findings demonstrate that the introduction of the dual-path reconstruction strategy leads to notable improvements in evaluation metrics across the four datasets while incurring minimal increases in parameter count and computational cost. Particularly noteworthy are the significant enhancements observed on Set14 and Urban100 datasets, both achieving a notable improvement of 0.04 dB. This strategy effectively preserves the low-frequency information and enhances the overall performance of the model.

### 3.13. Trade-off between Performance and Efficiency

In the experimental study of DLKL model ablation, we fine-tuned several core parameters and mechanisms, including LKA convolutional kernel size, number of MAFE (Multi-Attention Feature Extractor), number of channels, and incorporated LKA and ECA (Enhanced Spatial Attention) mechanisms. This initiative effectively reduces the number of model parameters and computational complexity, while maintaining excellent performance metrics. Specifically, as shown in Table 5, by adjusting the LKA configurations to 7-9-1 and 5-7-1, the number of parameters is reduced from 904.38K to 864.25K, and the FLOPs are reduced from 133.47 G to 127.35 G. Despite the slight decrease in the PSNR on the Urban100 dataset, the model training and inference process is significantly accelerated, which demonstrates the highly efficient computational capability. Further analyzing the data in Table 6, it is found that increasing the number of MAFEs and the number of channels can improve the model performance, but it is accompanied by an increase in computational complexity. After comprehensive consideration, we choose the configuration of six MAFEs with 64 channels to achieve the optimal balance of performance and computational efficiency. Table 7 demonstrates the significant enhancement of model performance by the LKA and ECA mechanisms, especially the excellent performance on PSNR and SSIM, which fully verifies the key role of these mechanisms in enhancing the model to capture long-range dependence and detail information. In addition, the comparative study in Table 8 emphasizes that the MAFE module achieves higher reconstruction accuracy than other feature extraction modules while maintaining lower parameter cost. Finally, Table 9 reveals the positive role of the dual-path reconstruction strategy in preserving low-frequency information and improving the overall reconstruction quality. With these optimizations, the DLKL model successfully achieves an effective balance between performance and computational efficiency.

## 4. Conclusions

In this paper, a novel Dual-path Large Kernel Learning (DLKL) mechanism is innovatively introduced, leading to the construction of a lightweight SISR model. This model effectively addresses many issues faced by traditional deep learning models, such as excessive parameters, long computation time, and difficulties in real-world deployment. By ingeniously integrating multiscale and large kernel decomposition mechanisms, DLKL successfully establishes robust long-range correlations between different regions while finely preserving intricate local detail information. Additionally, the performance of the network is further enhanced by improving the channel attention mechanism, enabling selective focus on key information within different channels. This design not only ensures compact parameter counts and efficient computational capabilities but also significantly enhances modeling expressiveness and reconstruction performance. Extensive experimental validation demonstrates the outstanding performance of DLKL. Compared with the state-of-the-art lightweight SISR methods like DLSR, DLKL achieves improvements of 0.15 dB and 0.0045 in PSNR and SSIM on the Urban100 dataset, respectively. This achievement fully demonstrates the superior performance of DLKL in terms of fewer parameters and computational resources.

While the information flow topology based on Dual-path Large Kernel Learning has shown some achievements in the super-resolution task, we acknowledge that it may not represent the ultimate optimization solution. Hence, in our future research, we aim to explore more efficient information flow typologies and refine large kernel learning strategies to attain substantial enhancements in system performance and further optimization.

## Figures and Tables

**Table 1:** Quantitative tests: The average PSNR and SSIM values for SISR results at a scale of ×4 on the datasets: Set5, Set14, BSD100, and Urban100. To assess the DLKL model’s performance, it is compared against other state-of-the-art methods, emphasizing the best results in **bold** and the second-best results with underline, respectively. The notation “-” indicates that the pertinent data are absent in instances where the authors have not released their source code publicly or in officially published papers.

Model	Scale	FLOP	Set5 [23]	Set14 [24]	BSD100 [25]	Urban100 [26]
			PSNR/SSIM	PSNR/SSIM	PSNR/SSIM	PSNR/SSIM
SRCNN (TPAMI 2014) [9]	×2	52.7 G	36.66/0.9542	32.43/0.9073	31.34/0.8879	29.50/0.8953
LapSRN (TPAMI 2017) [27]		29.9 G	37.52/0.9590	33.08/0.9130	31.80/0.8950	30.41/0.9100
IMDN (ACM MM 2019) [28]		186.7 G	38.00/0.9604	33.63/0.9173	32.19/0.8994	32.17/0.9283
SMSR (CVPR 2021) [29]		131.6 G	38.00/0.9601	33.64/0.9173	32.17/0.8990	32.19/0.9284
ShuffleMixer (NeurIPS 2022) [30]		91.0 G	38.01/0.9606	33.63/0.9180	32.17/0.8995	31.89/0.9257
LBNet (IJCAI 2022) [31]		-	38.05/0.9607	33.65/0.9177	32.15/0.8994	32.30/0.9291
ARRFN (NC 2022) [32]		-	38.01/0.9603	33.66/0.9174	32.20/0.8999	32.37/0.9295
LatticeNet (TPAMI 2023) [33]		169.0 G	38.06/0.9607	33.70/**0.9187**	32.20/0.8999	32.25/0.9302
PILN (TII 2023) [34]		-	38.08/0.9607	**33.72**/0.9181	**32.23**/0.9003	32.38/0.9306
DLSR (TCSVT 2023) [35]		-	38.04/0.9606	33.67/0.9183	32.21/0.9002	32.26/0.9297
MICU (ESWA 2024) [36]		-	37.93/0.9601	33.63/0.9170	32.17/0.8987	32.09/0.9271
HAFRN (TCE 2024) [37]		-	38.05/0.9606	33.66/**0.9187**	32.21/0.8999	32.20/0.9289
LBRN (ESWA 2024) [38]		-	38.08/0.9608	33.57/0.9173	**32.23**/**0.9005**	32.35/0.9303
Our DLKL		174.1 G	**38.12**/**0.9614**	33.71/**0.9187**	**32.23**/0.9004	**32.47**/**0.9315**
SRCNN (TPAMI 2014) [9]	×3	52.7 G	32.75/0.9090	29.30/0.8219	28.41/0.7863	26.25/0.7989
LapSRN (TPAMI 2017) [27]		-	33.82/0.9232	29.87/0.8324	28.82/0.7980	27.07/0.8281
IMDN (ACM MM 2019) [28]		84.0 G	34.36/0.9270	30.32/0.8417	29.09/0.8046	28.17/0.8519
SMSR (CVPR 2021) [29]		67.8 G	34.40/0.9270	30.33/0.8412	29.10/0.8050	28.25/0.8536
ShuffleMixer (NeurIPS 2022) [30]		43.0 G	34.40/0.9272	30.37/0.8423	29.12/0.8051	28.08/0.8498
LBNet (IJCAI 2022) [31]		-	34.47/0.9277	30.38/0.8417	29.13/0.8061	**28.42**/0.8559
ARRFN (NC 2022) [32]		-	34.38/0.9272	30.36/0.8422	29.09/0.8050	28.22/0.8533
LatticeNet (TPAMI 2023) [33]		76.0 G	34.40/0.9272	30.32/0.8416	29.10/0.8049	28.19/0.8513
PILN (TII 2023) [34]		-	34.39/0.9269	30.34/0.8415	29.08/0.8048	28.09/0.8500
DLSR (TCSVT 2023) [35]		-	34.49/0.9279	30.39/0.8428	29.13/0.8061	28.26/0.8548
MICU (ESWA 2024) [36]		-	34.38/0.9274	30.35/0.8419	29.10/0.8048	28.14/0.8518
HAFRN (TCE 2024) [37]		-	34.45/0.9276	30.40/0.8433	29.12/0.8058	28.16/0.8528
LBRN (ESWA 2024) [38]		-	34.43/0.9276	30.39/0.8429	29.13/0.8059	28.29/0.8545
Our DLKL		101.3 G	**34.57**/**0.9289**	**30.45**/**0.8438**	**29.16**/**0.8065**	28.37/**0.8569**
SRCNN (TPAMI 2014) [9]	×4	52.7 G	30.48/0.8628	27.50/0.7503	26.90/0.7110	24.53/0.7212
LapSRN (TPAMI 2017) [27]		149.4 G	31.54/0.8850	28.19/0.7720	27.32/0.7280	25.21/0.7560
IMDN (ACM MM 2019) [28]		48.0 G	32.21/0.8948	28.58/0.7811	27.56/0.7353	26.04/0.7838
SMSR (CVPR 2021) [29]		41.6 G	32.12/0.8932	28.55/0.7808	27.55/0.7351	26.11/0.7868
ShuffleMixer (NeurIPS 2022) [30]		28.0 G	32.21/0.8953	28.66/0.7827	27.61/0.7366	26.08/0.7835
LBNet (IJCAI 2022) [31]		-	32.29/0.8960	28.68/0.7832	27.62/0.7382	26.27/0.7906
ARRFN (NC 2022) [32]		-	32.22/0.8952	28.60/0.7817	27.57/0.7355	26.09/0.7858
LatticeNet (TPAMI 2023) [33]		43.0 G	32.18/0.8943	28.61/0.7812	27.57/0.7355	26.14/0.7844
PILN (TII 2023) [34]		-	32.22/0.8949	28.62/0.7813	27.59/0.7365	26.19/0.7878
DLSR (TCSVT 2023) [35]		20.41 G	32.33/0.8963	28.68/0.7832	27.61/0.7374	26.19/0.7892
MICU (ESWA 2024) [36]		-	32.21/0.8945	28.65/0.7820	27.57/0.7359	26.15/0.7872
HAFRN (TCE 2024) [37]		-	32.24/0.8953	28.60/0.7816	27.58/0.7365	26.02/0.7849
LBRN (ESWA 2024) [38]		-	32.33/0.8964	28.62/0.7826	27.60/0.7377	26.17/0.7882
Our DLKL		66.1 G	**32.39**/**0.8976**	**28.71**/**0.7853**	**27.65**/**0.7386**	**26.34**/**0.7937**

**Table 2:** MOS comparisons. The top 5 algorithms for scale factors ×4 on datasets Set5, Set14, BSD100, and Urban100. Our DLKL is highlighted in **black bold**.

Dataset	Scale	Top 5 Algorithms
Set5 [23]	×4	**DLKL** > LBNet > LatticeNet> LAMRN > ARRFN
Set14 [24]	×4	LatticeNet > **DLKL** > LAMRN > IMDN > LBNet
BSD100 [25]	×4	**DLKL** > LAMRN > ARRFN > LatticeNet> LBNet
Urban100 [26]	×4	**DLKL** > LBNet > ARRFN> LAMRN > LatticeNet

**Table 3:** The average PSNR and SSIM values for SISR results at a scale of ×4 on the datasets: Set5. To assess the DLKL model’s performance, it is compared against other state-of-the-art transformer methods, emphasizing the best results in **bold**.

Transformer-Based Methods	Scale	Para	Set5 [23]
LBNet (IJCAI 2022) [31]	×4	**742 K**	32.29/0.8960
ESRT (CVPR 2022) [40]	×4	751 K	32.19/0.8947
NGswin (CVPR 2023) [41]	×4	1019 K	32.33/0.8963
DLKL	×4	864 K	**32.39**/**0.8976**

**Table 4:** Comparisons on the model’s inference speed (ms).

Method	Speed	Method	Speed	Method	Speed	Method	Speed
SRCNN [9]	297	LapSRN [27]	189	EDSR-BASE [11]	315	IMDN [28]	38
SMSR [29]	309	ARRFN [32]	234	LBNet [31]	679	DLKL	142

**Table 5:** Effects of the LKA scale and multibranch mechanism on the performance of DLKA. The strategy used in this article is highlighted in **bold**, which is a comprehensive consideration of efficiency and performance. Specially, ✓ means the method is used, × means the method is not used.

LKA 3-5-1	LKA 5-7-1	LKA 7-9-1	Para	FLOP	Set5 [23] PSNR/SSIM	Set14 [24] PSNR/SSIM	BSD100 [25] PSNR/SSIM	Urban100 [26] PSNR/SSIM
✓	×	×	767.96 K	113.69 G	37.93/0.9587	33.43/0.9152	32.08/0.8975	31.74/0.9237
×	✓	×	778.20 K	115.20 G	37.94/0.9592	33.46/0.9159	32.10/0.8978	31.74/0.9238
×	×	✓	791.01 K	117.31 G	37.93/0.9584	33.44/0.9154	32.09/0.8977	31.73/0.9234
✓	✓	×	837.08 K	123.73 G	37.97/0.9598	33.50/0.9168	32.12/0.8983	31.80/0.9243
×	✓	✓	**864.25 K**	**127.35 G**	**37.98**/**0.9601**	**33.51**/**0.9171**	**32.11**/**0.8981**	**31.78**/**0.9238**
✓	×	✓	853.47 K	125.84 G	37.96/0.9601	33.50/0.9166	32.10/0.8976	31.77/0.9235
✓	✓	✓	904.38 K	133.47 G	37.98/0.9604	33.52/0.9176	32.11/0.8984	31.80/0.9246

**Table 6:** Comparison of model complexity and performance metrics: Average PSNR/SSIM for ×2 SISR results on benchmark datasets, where MAFEn and Chan indicate the number of MAFE and channels, respectively. Para denotes the number of model parameters, and FLOP refers to the quantity of floating-point operations. The strategy used in this article is highlighted in **bold**, which is a comprehensive consideration of efficiency and performance.

MAFEn	Chan	Para	FLOP	Set5 [23] PSNR/SSIM	Set14 [24] PSNR/SSIM	BSD100 [25] PSNR/SSIM	Urban100 [26] PSNR/SSIM
4	64	0.84 M	123.78 G	38.06/0.9603	33.61/0.9160	32.18/0.8994	32.15/0.9281
4	96	1.86 M	274.45 G	38.13/0.9617	33.71/0.9186	32.24/0.9006	32.40/0.9304
6	64	**1.18 M**	**174.1 G**	**38.12**/**0.9614**	**33.71**/**0.9187**	**32.23**/**0.9004**	**32.47**/**0.9315**
6	96	2.62 M	386.26 G	38.16/0.9621	33.83/0.9197	32.29/0.9011	32.60/0.9327

**Table 7:** Impact of LKA and ECA on MAFE Performance: Average PSNR/SSIM for ×2 SISR on Benchmarks, where Para denotes the number of model parameters, and FLOP refers to the quantity of floating-point operations. The strategy used in this article is highlighted in **bold**, which is a comprehensive consideration of efficiency and performance. Specially, ✓ means the method is used, × means the method is not used.

LKA	ECA	Para	FLOP	Set5 [23] PSNR/SSIM	Set14 [24] PSNR/SSIM	BSD100 [25] PSNR/SSIM	Urban100 [26] PSNR/SSIM
×	×	743.92 K	109.99 G	37.81/0.9573	33.34/0.9142	32.03/0.8969	31.62/0.9213
✓	×	837.08 K	123.73 G	37.97/0.9598	33.50/0.9168	32.12/0.8983	31.80/0.9243
×	✓	743.93 K	110.03 G	37.84/0.9576	33.40/0.9149	32.06/0.8972	31.72/0.9225
✓	✓	**837.09 K**	**123.77 G**	**37.99**/**0.9602**	**33.51**/**0.9169**	**32.15**/**0.8987**	**31.91**/**0.9255**

**Table 8:** Comparison of feature extraction blocks at scale factor 2, where Number represents the number of base blocks, Chan represents the number of channels, Para denotes the number of model parameters, and FLOP refers to the quantity of floating-point operations. The strategy used in this article is highlighted in **bold**, which is a comprehensive consideration of efficiency and performance.

Block	Number	Chan	Para	FLOP	Set5 [23] PSNR/SSIM	Set14 [24] PSNR/SSIM	BSD100 [25] PSNR/SSIM	Urban100 [26] PSNR/SSIM
Resblock [42]	12	64	1.04 M	153.48 G	37.74/0.9587	33.25/0.9139	31.95/0.8963	31.07/0.9162
RRDB [46]	4	64	0.89 M	131.73 G	37.87/0.9598	33.38/0.9155	32.05/0.8972	31.48/0.9193
RCAB [13]	4	64	0.74 M	66.54 G	37.77/0.9591	33.21/0.9132	31.92/0.8960	31.09/0.9165
MAB [47]	14	90	1.08 M	158.50 G	37.94/0.9601	33.48/0.9162	32.15/0.8986	31.79/0.9232
DLKL	4	64	**0.83 M**	**123.77 G**	**37.99**/**0.9604**	**33.51**/**0.9165**	**32.15**/**0.8987**	**31.91**/**0.9254**

**Table 9:** Impact of dual-path reconstruction on ×2 SISR performance: Average PSNR/SSIM on Benchmarks. The best results are highlighted in **bold**. Specially, ✓ means the method is used, × means the method is not used.

Dual Path	Set5 [23] PSNR/SSIM	Set14 [24] PSNR/SSIM	BSD100 [25] PSNR/SSIM	Urban100 [26] PSNR/SSIM
×	37.99/0.9602	33.51/0.9169	32.15/0.8987	31.91/0.9255
✓	**38.00**/**0.9605**	**33.55**/**0.9175**	**32.16**/**0.8993**	**31.95**/**0.9264**

## Data Availability

The data presented in this study are available on request from the corresponding author.
